# Evaluation of a multiplex PCR assay for detection of respiratory viruses and *Mycoplasma pneumoniae* in oropharyngeal swab samples from outpatients

**DOI:** 10.1002/jcla.23032

**Published:** 2019-10-19

**Authors:** Ying Zhang, Lan Cao, Zhi Xu, Pingting Zhu, Bing Huang, Kuibiao Li, Yang Xu, Zhoubin Zhang, Yong Wu, Biao Di

**Affiliations:** ^1^ Guangzhou Center for Disease Control and Prevention Guangzhou China; ^2^ Ningbo Health Gene Technologies Co., Ltd Ningbo China

**Keywords:** acute respiratory infections, influenza virus A, multiplex PCR, oropharyngeal swab, respiratory virus

## Abstract

**Background:**

Respiratory viruses, such as influenza viruses, initially infect the upper airways but can manifest as severe lower respiratory tract infections in high‐risk patients with significant morbidity and mortality. For syndromic diagnosis, several multiplex nucleic acid amplification tests have been developed for clinics, of which SureX 13 Respiratory Pathogen Multiplex Kit (ResP) can simultaneously detect 13 pathogens directly from airway secretion specimens. The organisms identified are influenza virus A, influenza virus A pdmH1N1 (2009), influenza virus A H3N2, influenza virus B, adenovirus, boca virus, rhinovirus, parainfluenza virus, coronavirus, respiratory syncytial virus, human metapneumovirus, *Mycoplasma pneumoniae,* and *Chlamydia*.

**Methods:**

This study provides performance evaluation data of this assay by comparing with pathogen‐specific PCRs from oropharyngeal swab samples.

**Results:**

Ten pathogens were detected in this assay, of which rhinovirus, adenovirus, and influenza virus A pdmH1N1 (2009) were the most common. The overall agreement between the ResP and the comparator tests was 93.8%. The ResP demonstrated 86.5% agreement for positive results and 97.8% agreement for negative results.

**Conclusion:**

The ResP assay demonstrated a highly concordant performance comparing with pathogen‐specific PCRs for detection of respiratory pathogens in oropharyngeal swabs from outpatients and could aid in the diagnosis of respiratory infections in a variety of clinical scenarios.

## 1 INTRODUCTION

Acute respiratory infections (ARIs) are common and contribute significantly to morbidity and mortality. They are the leading causes of outpatient visits and hospitalizations in all age groups, especially for children under 5 years of age.^1^ Most ARIs in children and outpatients are caused by nine common respiratory viruses, including respiratory syncytial virus (RSV), influenza virus A, influenza virus B, rhinovirus, adenovirus, parainfluenza virus, coronavirus, human metapneumovirus, and boca virus^2^, ^3^ Additionally, atypical pathogens, such as *Mycoplasma pneumoniae,* are also major causes of ARIs in children. The symptoms caused by these pathogens are largely similar, thus definitive diagnosis requires effective laboratory testing. By using multiplex assay targeting these pathogens, early diagnosis can be made in a timely manner. Consequential antimicrobial or antiviral therapy may thus be administrated promptly and appropriately.^4^ Most importantly, the early diagnosis of influenza viruses, which are contagious, is beneficial for early isolation of patients, thus reducing the spread of influenza viruses.

The routine clinical laboratory testing for respiratory viruses is largely conducted by direct fluorescent‐antibody assays and rapid antigen tests in China. Given the poor sensitivity and complicated manual operation, these methods have been gradually replaced by nucleic acid amplification tests (NAATs), which are more sensitive and more specific. However, majority of the NAAT kits are based on real‐time polymerase chain reaction (PCR), which can only detect one or two pathogens of ARIs within a single tube, thus are not syndromic testing.^5^ The clinical and economic impacts of syndromic testing for respiratory pathogens have been evaluated in several studies. Overall, the implementation of syndromic testing can decrease the time of diagnosis,^4^ decreased healthcare resource utilization,^6^ decrease inpatient length of stay and time in isolation,^7^ and improve antiviral use for influenza virus‐positive patients.^8^

SureX 13 Respiratory Pathogen Multiplex Kit (ResP) is a syndromic multiplex molecular test for simultaneous detection of 13 pathogens in a single tube. The aim of this study was to evaluate the application of the ResP for detection of respiratory pathogens in outpatients with flu‐like manifestations.

## 2 MATERIALS AND METHODS

### 2.1 Samples

The inclusion criteria for this study were as follows: (a) patients admitted to hospitals between Feb. 2017 and Aug. 2018; (b) oropharyngeal swabs were collected from hospitals and Centers for Disease Control in Guangzhou; (c) patients had the following flu‐like manifestations: (a) fever (>38°C); (b) cough or sore throat. After sampling, specimens were kept in 4°C and transferred to the laboratory for testing within one week.

### 2.2 Nucleic acid extraction

The specimen was shaken vigorously for 5 minutes in phosphate‐buffered saline solution, centrifuged at 9.6 *g* for 20 minutes, and the supernatant was aspirated. About 50 µL of RNA was extracted from 140 µL supernatant using the QIAamp Viral RNA extraction kit (QIAGEN, Hilden, Germany), according to the manufacture's instruction and was stored at −80°C.

### 2.3 Detection of influenza viruses

Influenza virus nucleic acid detection was performed by Influenza A/B Influenza Virus Nucleic Acid Detection Kit (Cat. No. DA‐BN147, Daan Gene). Positive samples were further tested for influenza virus A pdmH1N1 (2009) and seasonal influenza virus H3N2 using a separate kit (Cat. No. JC10209, Daan Gene). Both tests were carried out on ABI Quant Studio 7 System (Thermo Fisher Scientific) according to the instructions. A typical S amplification curve and Cq value ≤35.0 were determined positive.

### 2.4 Detection of other respiratory pathogens

For influenza virus‐negative samples, more PCR tests were performed to detect the following pathogens: adenovirus (ADV), bocavirus (BOV), human rhinovirus (HRV), parainfluenza virus (PIV), human metapneumovirus (HMPV), *Mycoplasma pneumoniae* (MP), and respiratory syncytial virus (RSV), using corresponding NAAT kits from Daan Gene. All tests were carried out on ABI Quant Studio 7 System (Thermo Fisher Scientific) according to the instructions. A typical S amplification curve and Cq value ≤38.0 were determined positive.

### 2.5 Multiplex detection of respiratory pathogens

The nucleic acid was subjected to multiplex amplification for all specimens using SureX 13 Respiratory Pathogen Multiplex Detection Kit (Cat. No. 1 060 144, Ningbo Health Gene Technology) on ABI GeneAmp PCR System 9700 (Thermo Fisher Scientific). The 13 respiratory pathogens were as following: influenza A virus, influenza A virus H1N1 (2009), seasonal H3N2 influenza virus, influenza B virus, adenovirus, boca virus, rhinovirus, parainfluenza virus, *chlamydia,* human metapneumovirus, *Mycoplasma pneumoniae*, coronavirus, and respiratory syncytial virus. The PCR product was subjected to capillary electrophoresis using GenomeLab™ GeXP Genetic Analysis System (Beckman Coulter) according to the instructions. Each pathogen, if detectable, produced a distinctive fragment size after PCR amplification. The results of fragment analysis were used to determine the outcomes of testing. In brief, if the peak height of a targeted fragment size is lower than the lower peak of the signal standard, the targeted pathogen is determined negative; if the peak height of a targeted fragment size is higher than the higher peak of the signal standard, the targeted pathogen is determined positive; if the peak height of a targeted fragment size is between the higher and the lower peaks of the signal standard, the targeted pathogen is determined uncertain and the test should be repeated.

### 2.6 Statistical analysis

The results were analyzed using EXCEL2007. The Cohen's kappa statistics were calculated to measure the agreement between pathogen‐specific PCRs and multiplex PCR results (<0 = poor, 0‐0.2 = slight, 0.21‐0.4 = fair, 0.41‐0.6 = moderate, 0.61‐0.8 = substantial, and 0.81‐1 = almost perfect).^9^ *P* value was calculated by CHITEST, and *P* < .05 indicates statistical significance.

## 3 RESULTS

A total of 420 oropharyngeal swabs were enrolled from 10 hospitals and 10 CDCs in Guangzhou from 2017 to 2018. Samples were collected from a wide range of ages, with the average age of 27.2 (Table 1). About 55% specimens were from male.

**Table 1:** Samples enrolled in this study

Item	n	Average age (y)
Gender		
Male	231	26.5
Female	189	28.2
Age group		
<5	111	2.1
5 ~ 18	92	8.8
18 ~ 60	150	36.4
>60	67	73.8
Total	420	27.2

**Figure 1:**
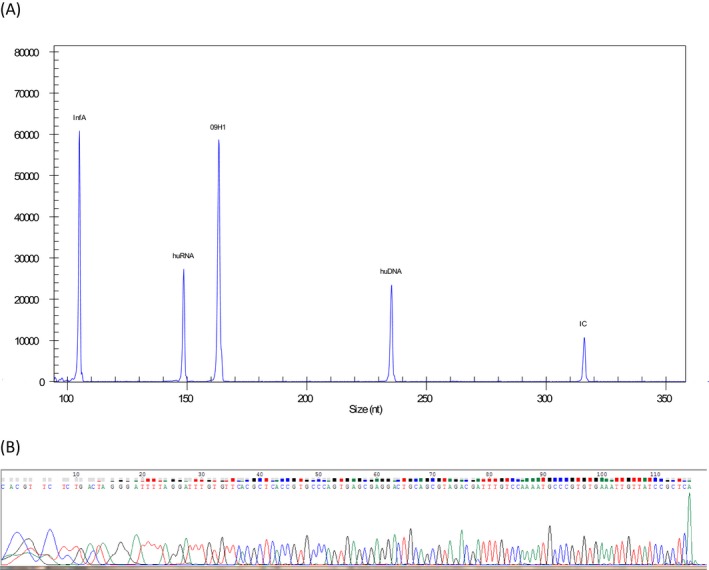
An example of influenza virus A pdmH1N1 (2009)‐positive result from the multiple PCR assay ResP. A, X‐axis represents the sizes of amplification products, and Y‐axis represents the signal strength. InfA, influenza virus A; 09H1, influenza virus A pdmH1N1 (2009); huRNA, human RNA; huDNA, human DNA; IC, internal control; B, Sanger sequencing result of partial sequence of influenza virus A pdmH1N1 (2009)

**Figure 2:**
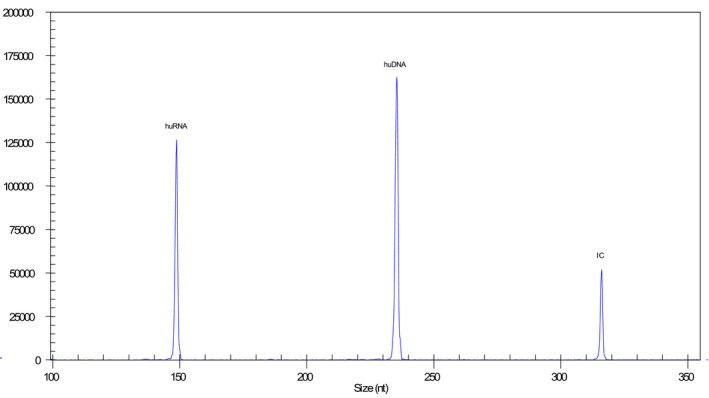
A negative result from the multiple PCR assay ResP. X‐axis represents the sizes of amplification products and Y‐axis represents the signal strength. huRNA, human RNA; huDNA, human DNA; IC, internal control

A pathogen‐positive result was determined when the pathogen‐specific fragment(s) was positive, as shown in Figure 1. A negative result was determined when none of the 13 pathogen‐specific fragment was positive, while the controls (huDNA, huRNA, and IC) were positive (Figure 2). In this study, the ResP detected positive results in 141 samples, accounting for 33.6%, while the comparator tests detected positive results in 127 samples, with positive rate 30.2%. Among the detected pathogens, rhinovirus was the most common, followed by adenovirus and influenza virus A pdmH1N1 (2009) (Table 2). Of the 420 specimens, the ResP yielded consistent positive results in 121 specimens (86.5%, 121/141), and consistent negative results in 273 specimens (97.8%, 273/279) comparing with pathogen‐specific PCRs, leading to an overall agreement of 93.8%.

**Table 2:** Pathogens detected by ResP and pathogen‐specific PCRs

Pathogen	ResP	Pathogen‐specific PCRs	Kappa	*P*
Rhinovirus	34	28	0.82	＜.01
Adenovirus	30	28	0.81	＜.01
Influenza virus A pdmH1N1 (2009)	30	30	1.00	＜.01
Respiratory syncytial virus	16	14	0.93	＜.01
Influenza virus B	12	11	0.96	＜.01
Human metapneumovirus	10	7	0.7	＜.01
Parainfluenza virus	7	6	N/A	N/A
*Mycoplasma pneumoniae*	7	6	N/A	N/A
Boca virus	4	4	N/A	N/A
Influenza virus A H3N2	1	1	N/A	N/A
Total	151	135	0.86	＜.01

Abbreviation: N/A, not available.

No specimen was detected positive with coronavirus or *Chlamydia*. In six of the ten detected pathogens, the Cohen's kappa values were over 0.8 with *P* value <.01 (Table 2). The lowest kappa (0.70) was observed on human metapneumovirus.

## 4 DISCUSSION

Multiplex PCR‐based NAATs have been increasingly used for syndromic diagnosis, due to their high throughput, high sensitivity, high specificity, cost‐effectiveness, and great clinical significance.^10^, ^11^, ^12^ The ResP assay is based on multiplex PCR amplification and capillary electrophoretic separation of PCR amplicons by length. This technique has been used for pathogen detection and subtype classification of pediatric acute lymphoblastic leukemia.^13^, ^14^ By comparing the results with a standard size marker of targeted pathogens, pathogens in samples can be separated and identified as expected.^15^ The subtypes of most viruses were not designed to be further distinguished by this assay, except for influenza virus A. The influenza virus A pdmH1N1 (2009) and H3N2 are the two subtypes which are most popular in China recently. Therefore, a patient whose specimen is positive for influenza virus A but negative for influenza virus A pdmH1N1 (2009) or H3N2 is probably infected by an uncommon influenza virus A, such as H7N9, H5N1, H5N6 avian influenza virus A^16^, ^17^, ^18^ and has to be immediately quarantined once it is confirmed. It should be noted that hospitals, not CDCs, are the first to reach such patients, so this assay helps hospitals identifying such high‐risk patients and make appropriate quarantine measurement in a timely manner to control further spread of avian influenza A virus.

This assay has previously been clinically applied to detection of respiratory pathogens in hospitalized children suffered with community‐acquired pneumonia (CAP)^14^ or lower respiratory tract infections.^19^ The assay was evaluated by comparing with Sanger sequencing, showing great performance with 100% positive prediction value (PPV) and 99.85% negative prediction value (NPV).^20^ To our knowledge, this is the first study evaluating the performance of the ResP in oropharyngeal swab specimens from outpatients with ARIs.

Our study showed almost perfect kappa statistics for the ResP on rhinovirus, adenovirus, influenza virus A pdmH1N1(2009), respiratory syncytial virus, and influenza virus B, suggesting that the performance of ResP on these viruses was as effective as pathogen‐specific PCRs. On human metapneumovirus, the kappa statistics were lower than 0.8, presumably due to the small number of positive cases. Overall, this assay demonstrated 86.5% PPV and 97.8% NPV. This work suggested that the performance of ResP was sufficient enough be used for respiratory pathogen identification in outpatients with flu‐like manifestations.

The major limitation of this study is the small number of human metapneumovirus, parainfluenza virus, *Mycoplasma pneumoniae*, boca virus, influenza virus A H3N2, coronavirus, and *Chlamydia.* Further investigation is needed to evaluate the performance of ResP on these pathogens.

In conclusion, the performance of ResP showed a high‐degree agreement with pathogen‐specific PCRs in oropharyngeal swabs from outpatients. The implementation of ResP may facilitate the diagnosis of respiratory infections in a variety of clinical scenarios.

## FUNDING INFORMATION

The study was supported by the grants from the National Science and Technology Major Project of the Ministry of Science and Technology of China (2017ZX10103011), the Project for Key Medicine Discipline Construction of Guangzhou Municipality (2017‐2019‐07), Science and Technology Program of Guangdong Health Department (A2017489), Science and Technology Program of Guangzhou Health Department (20181A011052).
